# Chemistry of resistivity changes in TiTe/Al_2_O_3_ conductive-bridge memories

**DOI:** 10.1038/s41598-018-36131-7

**Published:** 2018-12-18

**Authors:** M. Kazar Mendes, E. Martinez, J. M. Ablett, M. Veillerot, R. Gassilloud, M. Bernard, O. Renault, J. P. Rueff, N. Barrett

**Affiliations:** 1 Univ. Grenoble Alpes, CEA, LETI, 38000 Grenoble, France; 2 Synchrotron SOLEIL, l’Orme des Merisiers, Saint-Aubin, F-91192 Gif-sur-Yvette Cedex, France; 3 Sorbonne Université, UPMC Univ Paris 06, CNRS, UMR 7614, Laboratoire de Chimie Physique-Matière et Rayonnement, 75005 Paris Cedex 05, France; 4 SPEC, CEA, CNRS, Université Paris-Saclay, CEA Saclay, 91191 Gif-sur-Yvette, France

## Abstract

We report the chemical phenomena involved in the reverse forming (negative bias on top electrode) and reset of a TaN/TiTe/Al_2_O_3_/Ta memory stack. Hard X-ray photoelectron spectroscopy was used to conduct a non-destructive investigation of the critical interfaces between the electrolyte (Al_2_O_3_) and the TiTe top and Ta bottom electrodes. During reverse forming, Te accumulates at the TiTe/Al_2_O_3_ interface, the TiO_x_ layer between the electrolyte and the electrode is reduced and the TaO_x_ at the interface with Al_2_O_3_ is oxidized. These interfacial redox processes are related to an oxygen drift toward the bottom electrode under applied bias, which may favour Te transport into the electrolyte. Thus, the forming processes is related to both Te release and also to the probable migration of oxygen vacancies inside the alumina layer. The opposite phenomena are observed during the reset. TiO_x_ is oxidized near Al_2_O_3_ and TaO_x_ is reduced at the Al_2_O_3_/Ta interface, following the O^2−^ drift towards the top electrode under positive bias while Te is driven back into the TiTe electrode.

## Introduction

Resistive Random Access Memories (RRAM) are candidates for the next generation of non-volatile memories (NVMs) thanks to their switching properties^1,2^. Among them, conductive bridge random access memories (CBRAMs) are one promising solution to improve the cycling endurance while maintaining good scaling and high operation speed^3–5^. In these devices, an electrolyte (chalcogenide or a suitable oxide, for instance) is used as a dielectric layer whose resistive state is changed by the diffusion of cations from the active electrode, containing most often Ag or Cu^1,4,6^ to form a conducting bridge. Changes in resistivity are explained by the formation and partial destruction of Ag or Cu-rich filaments inside the electrolyte. The filament creation known as forming, is an irreversible process required to enable the write/erase (SET/RESET) cycles of the memory cell. The CBRAMs structures are characterized by higher resistance windows^7^, i.e. the ratio between the high (HRS) and low (LRS) resistance state, compared to transition metal oxide-based RRAMs (OxRRAMs), and also by lower programming currents.

Recently, devices containing an elemental semiconductor such as tellurium, operating with reduced currents and less retention failures of the LRS^8^ have drawn special attention. In these subquantum CBRAM cells, the filament is thought to contain tellurium, yielding a 1-atom conductance (G_1atom_), significantly reduced compared to standard CBRAMs and thus allowing low power operation. Subquantum CBRAMs based on an Al_2_O_3_ electrolyte and an active top electrode of a Te binary alloy such as ZrTe, TiTe and HfTe, are the most promising systems with cell operating current and power as low as 10 µA and 0.01 mW^8^. The origin of resistive switching in Te-based subquantum memories with ZrTe active electrode is attributed to the release of Te following Zr oxidation due to oxygen scavenging from the Al_2_O_3_ under the electric field^9^. Note that Te-filaments have also been observed in CBRAMs based on Ge_2_Sb_2_Te_5_ (GST) sandwiched between inert electrodes (Ti and Pt). In the case of GST containing a Te-rich interfacial region, the resistive switching is no longer related to the usual phase change mechanism. The LRS was shown to be semiconducting and due to Te ions migration and reduction at the opposite electrode^10^.

TiTe features a retention behaviour controlled by a similar critical conductance as that for ZrTe^8^. The investigation of this memory cell mechanism is important since not all binary alloys of Te favor the formation of a semiconductor filament. For example, in the case of the CuTe_x_ the filament is formed preferentially by Cu^11^. Therefore, it is necessary to understand the role of the metal and the semiconductor during the resistance switching for each Te-based electrode variant.

Here, we investigate the negative forming, also called reverse forming, in which a negative voltage is applied to the top electrode and the first reset step i.e. from LRS to HRS. The reverse forming was analysed in this case since the positive polarisation caused an irreversible switching of the TiTe/Al_2_O_3_ cell unlike the negative polarization that enabled the reset of the memory after the forming process. Negative forming was observed in several standard Cu-based CBRAMs structures^12–14^ and reported to be related to metal cations and/or oxygen vacancies (${{\rm{V}}}_{{\rm{o}}}^{\bullet \bullet }$). Compared to positive forming, the bottom electrode, here Ta, may also play a role in addition to the top TiTe electrode.

In these complex systems, the interfaces between the electrodes and the solid electrolyte is known to play a critical role^15^. Composition changes at these interfaces are very often observed, even for RRAMs showing filamentary-type switching. Regarding OXRRAMs, metals with high oxygen affinity (Ta, Ti…) are often used as electrodes. They form interfacial oxides that change the electrolyte composition by oxygen exchange. For example, in the ZrO_2_/Ti system, it has been shown that a TiO_x_ film is created at the interface which in turn reduces the oxide matrix to form ZrO_2−x_^16^. This is also the case for a metal in contact with its stoichiometric oxide, as reported for the Ta/Ta_2_O_5_ system where an intermediate TaO_2_ oxide is formed^17^. For CBRAMs, the typical interface interaction involves the metal oxidation with the insertion of oxidized ions in the electrolyte. For example, in the Cu/SiO_2_ system, Cu oxidation was found to provide Cu^2+^ ions diffusing from the top electrode inside the electrolyte and participating in the resistive switching^18^. Similar effects were also reported near the bottom electrode^19^. All these interfacial interactions may strongly influence the behavior of the memory cell and is also the case for Te-based subquantum CBRAMs in this paper.

More information about resistive swithing would facilitate the control and manufacturing of such memory cells. It is also necessary to better understand the cycling of the cell. Thus, not only the electroforming step^9^, but also the switching between the LRS to the HRS, known as the RESET step must be investigated. To study the resistive state-dependent chemistry in such Te-based CBRAMs requires advanced characterization techniques. X-ray photoelectron spectroscopy is particularly interesting because it provides information about the chemical states. Hard X-ray photoelectron spectroscopy (HAXPES) enables non-destructive analysis of the buried interfaces between realistic electrodes and insulating layer thanks to the much greater probing depth^20^. Another advantage of HAXPES compared to laboratory XPS is related to the high brightness offered by third generation synchrotron sources (10^21^–10^22^ photons/s/mm^2^/mrad^2^/0.1% BW). This can be used to highlight additional changes of the whole matrix accompanying the filament formation, in particular interface modifications. The technique has already been successfully used to investigate the redox reactions that occurs during the resistive switching of oxide-based resistive memories^21–23^ and CBRAMs^9^.

We have used HAXPES to study the physical chemistry of forming and resistive switching of TiTe/Al_2_O_3_/Ta CBRAMs. We compare results obtained for three samples: as-grown i.e. the pristine device with an HRS, formed i.e. the sample after the first transition between HRS and LRS and reset i.e. the sample after the first transition from LRS to HRS. The aim is to clarify the switching mechanisms and understand the chemical changes at both interfaces. As reported by Cho *et al*.^24^, the oxidation/reduction reactions at metal/oxide interfaces induce the formation of intermediate layers that can influence the resistive switching. Note that the forming process implies a negative bias on the top electrode and so strictly speaking is reverse forming, however, hereafter we will refer to the process simply as forming.

## Methods

**Figure 1:**
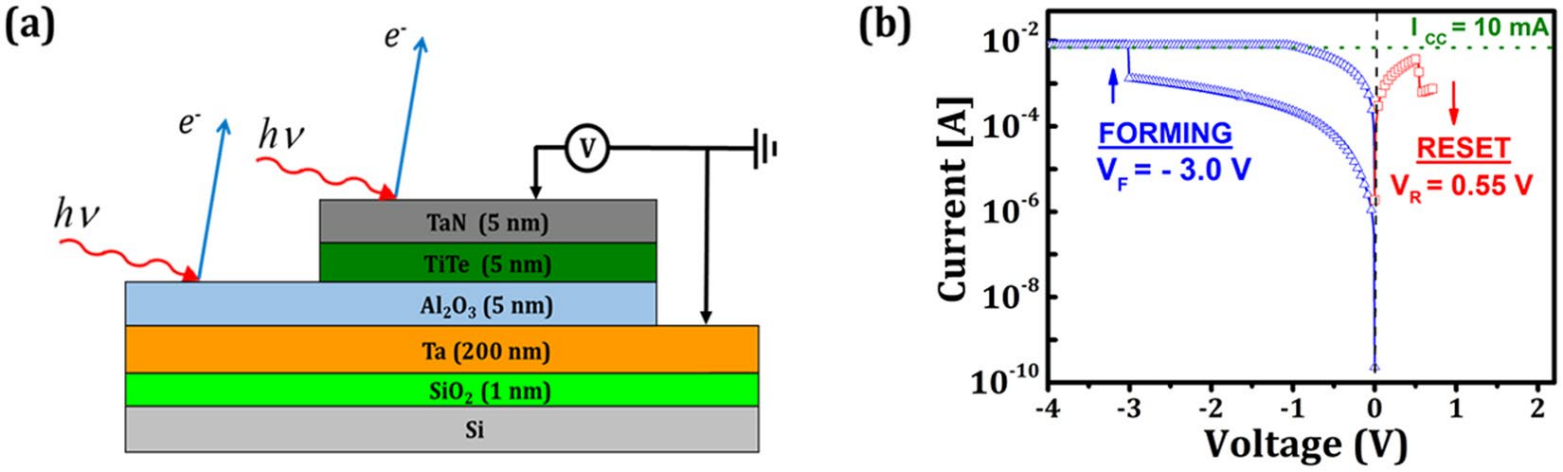
(**a**) Schematic of the TaN/TiTe/Al_2_O_3_/Ta device structure of the HAXPES measurements and schematic of the setup for electrical characterization (**b**) I-V curve for the forming and the reset of the TaN/TiTe/Al_2_O_3_/Ta stack.

The CBRAM is the TaN/TiTe/Al_2_O_3_/Ta structure (see Fig. 1(a)). The bottom electrode was a 200 nm-thick Ta layer, deposited on a 200 mm Si (100) wafer. The 5 nm TiTe and Al_2_O_3_ layers were grown by physical vapor deposition (PVD) at room temperature. The growth process was optimized to reach a Ti/Te stoichiometry of 40/60. The base pressure in the deposition chamber was 5 × 10^−4^ mbar. Finally, a 5-nm TaN capping layer was deposited by reactive sputtering at 50 °C to prevent oxidation of TiTe when exposed to air. The TaN and TiTe layers were deposited through a mask to define a 2 mm diameter top electrode.

The CBRAM structures were analysed in the as-grown state, after forming and after reset. The switching was performed in ambient atmosphere using a Keithley 2635B and the Ta bottom electrode was grounded. A linear voltage sweep was applied by an Au tip with minimum contact force on the TaN (Fig. 1(a)) at 0.1 V/s, between 0 and −4 V for the electroforming step and from 0 to 0.7 V for the reset step. A standard electrical connection was not used for the top electrode because the ultra-thin alumina layer can be easily short-circuited by mechanical or thermal stress. The electrical contact to the bottom electrode was made with a W tip. The compliance current of 10 mA (0.03 mA.cm^−2^) was chosen to avoid permanent breakdown of the oxide while inducing significant ionic diffusion, facilitating the detection^3^.

HAXPES analyses were performed at the GALAXIES beamline of the SOLEIL synchrotron^25^. All measurements were performed at room temperature. The TiTe electrode, the TiTe/Al_2_O_3_ interface, the thin oxide film (Al_2_O_3_) and the bottom Al_2_O_3_/Ta interface were probed by tuning the inelastic mean free path (λ) using photon energies of 6.9, 8 and 10 keV. The values of λ are estimated with the Tanuma equation^26^ for Al 1*s*, Ti 1*s*, Te 3*d*_3/2_ and Ta 3*d*_5/2_ photoelectrons and are given in Table 1. These values are obtained by averaging the IMFPs^27^ for each layer crossed by the photoelectrons during their transport toward the surface, weighted by the thickness of each layer (see Supplementary Information). The overall energy resolution (beamline and spectrometer) was 110, 160 and 210 meV for 6.9, 8 and 10 keV photon energies respectively.

**Table 1:** Inelastic mean free path for photon energy of 6.9, 8 and 10 keV.

	6.9 keV	8.0 keV	10 keV
	λ (nm)	λ (nm)	λ (nm)
Al 1*s*	8,5 ± 1.7	9.8 ± 1.9	12.4 ± 2.4
Ti 1*s*	3.7 ± 0.7	5.0 ± 1.0	8.0 ± 1.6
Te 3*d*_3/2_	9.8 ± 1.9	11.1 ± 2.2	13.7 ± 2.7
Ta 3*d*_5/2_	8.3 ± 1.6	9.7 ± 1.8	12.2 ± 2.3

The sampling depth is defined to be 1.5 λ, corresponding to 78% of total intensity.

The X-rays beam spot size on the sample was 500 × 30 µm^2^ (with an X-ray incidence angle of 10° with respect to the surface) and photoelectrons emitted at an angle of 80° with respect to the surface were collected with a hemispherical SCIENTA EW4000 electron analyzer. The binding energy was calibrated relative to the Au 4*f* level measured from a clean gold surface and also relative to the Ta 3*d*_5/2_ level measured on the Al_2_O_3_/Ta interface outside the device areas (as a stable internal reference). The deconvolution of the core level spectra was performed using the Casa XPS software, version 2.3.16 from Casa Software Ltd. Backgrounds for XPS spectra were of the Shirley type^28^. The oxide peaks were modeled using a convolution of Lorentzian (30%) and Gaussian (70%) functions while the metallic/semiconductor core lines were fitted using a Doniach-Sunjic function^29^. The peaks fit was made establishing constrains on components positions and fixing the separation between each chemical state. All the details regarding the binding energies used for fitting are presented in Table 2.

**Table 2:** Binding energies (eV) of the Ta 3*d*_5/2_, Ti 1*s*, Te 3*d*_3/2_ and Al 1*s* components measured for the as-grown, formed and reset samples^27^.

	Components	Binding energies (eV)		
		As-Grown	Formed	Reset
Ta 3*d*_5/2_	Ta	1731.5	1731.7	1731.7
	TaOx or TaN	1734.1	1734.2	1734.2
	Ta_2_O_5_	1735.8	1736.0	1736.0
Ti 1*s*	TiTe	4965.5	4965.7	4965.7
	TiO_x_	4966.9	4967.1	4967.1
	TiO_2_	4968.7	4968.9	4968.9
Te 3*d*_3/2_	TiTe	582.9	583.1	583.1
	Te	583.5	583.7	583.7
	TeO_x_	584.8	585.0	585.0
Al 1s	AlO_x_	1562.0	1562.2	1562.2
	Al_2_O_3_	1562.8	1563.0	1563.0

Time-of-flight secondary ion mass spectrometry (ToF-SIMS) was used to study the elemental distribution depth profiles and possible interdiffusion for as-grown, formed and reset samples. ToF-SIMS data were collected using an ION-TOF ToF-SIMS 5 instrument. The Bi_3_^+^ analysis beam generated by a bismuth source, was accelerated at 25 keV and rastered over an area of 80 × 80 μm. Cesium ions were used for sputtering, rastered over a 300 × 300 µm² area, at an impact energy of 500 eV. Both beams were incident at 45°. The intensities of the M^+^ ions and MCs^+^ clusters secondary ions (where M is the element to be analyzed and Cs^+^ is the bombarding ion) were used for the analysis.

## Results

### Electrical characterization

The I-V curves measured for the TaN/TiTe/Al_2_O_3_/Ta devices are shown in Fig. 1(b) for the forming and reset steps. During the forming step, the current increases slowly when applying the negative voltage and then abruptly at the forming voltage (*V*_f_) of 3.0 V to reach the 10 mA compliance current. During the reset step, a positive voltage is applied to the structure until reaching an abrupt decrease of the current at the reset voltage (V_R_) of 0.55 V.

The device resistance measured before and after forming are R_As-grown_ = 3.3 × 10^4^ Ω and R_Formed_ = 1.6 × 10^2^ Ω. The resistance ratio R_As-grown_/R_Formed_ = 2.1 × 10^2^ shows a change of two orders of magnitude in the device resistivity by the formation of a conductive path (or paths) in the electrolyte. The resistance measured after reset, R_Reset_ = 5.0 × 10^3^ Ω, confirms the return towards the high resistive state. The HRS/LRS ratio is therefore ~30.

The current - voltage (I-V) curves presented in Fig. 1(b) were measured for the sample after resetting. The I-V curve shown in Fig. S1 was measured on a different sample which had only undergone forming.

### Hard X-ray photoelectron spectroscopy

#### 6.9 keV

HAXPES was performed on the as-grown, formed and reset samples, and also on the bare Al_2_O_3_/Ta structure outside the top electrode in order to provide reference spectra for tantalum and aluminum (Fig. 1(a)). Each peak was normalized relative to the background intensity measured at high kinetic energy ensuring the removal of all geometrical effects and allowing a quantitative comparison of core levels intensities. We have estimated the recoil energy (δE) using δE = E_kin_ (m/M) where E_kin_ is the photoelectron kinetic energy, m is the electron mass and M the atomic mass^30^. The recoil energies for Al 1*s* (0.1 eV), Ti 1*s* (0.02 eV), Te 3*d*_3/2_ (0.03 eV), and Ta 3*d*_5/2_ (0.01 eV) core levels. Apart from the recoil of the Al 1s, all are less than the observed energy chemical shifts and can therefore be neglected.

**Figure 2:**
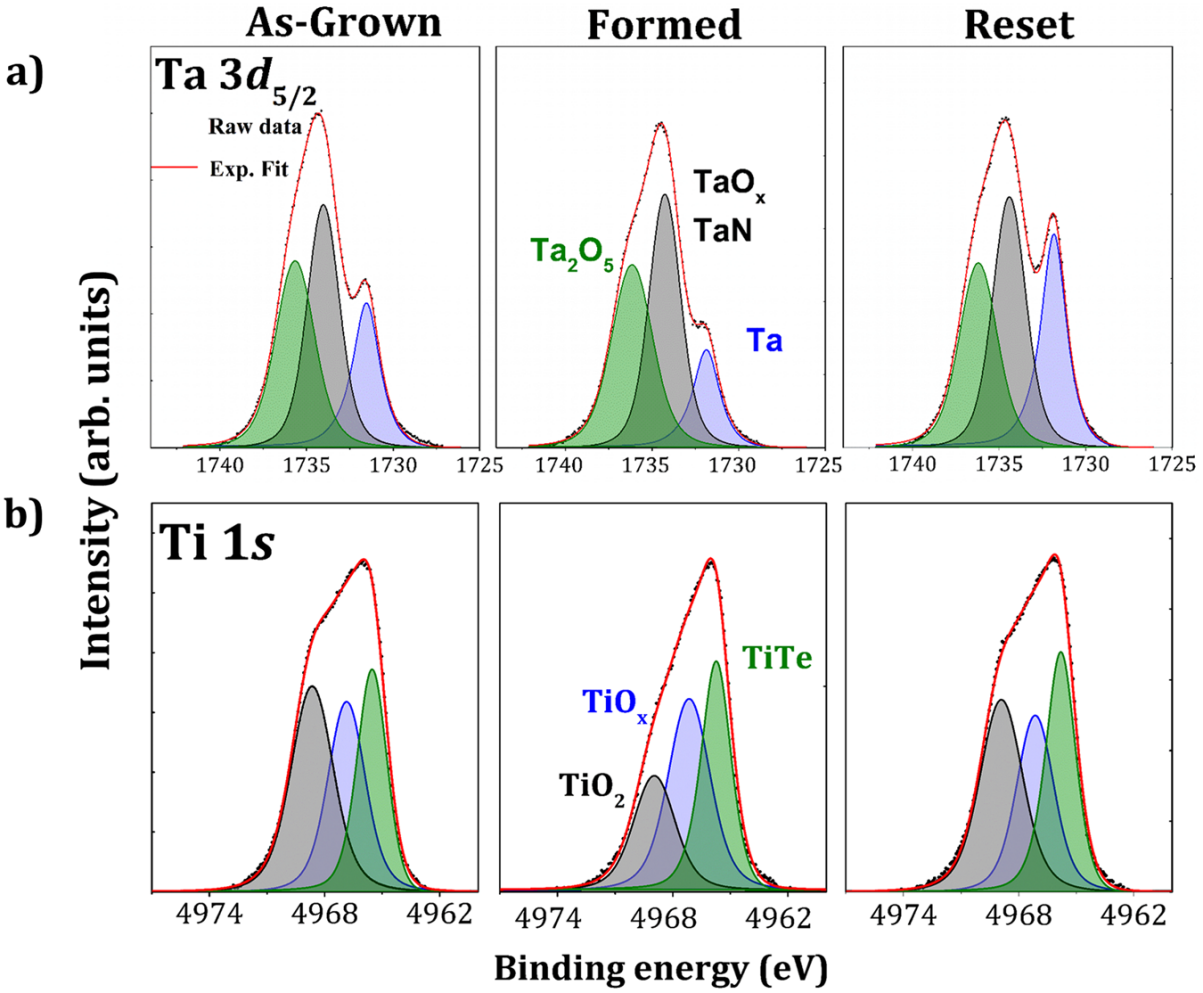
(**a**) Ta 3*d*_5/2_ and (**b**) Ti 1*s* core level peaks obtained by HAXPES at 6.9 keV on as-grown, formed and reset sample of the TaN/TiTe/Al_2_O_3_/Ta stack.

Electrode metal chemistry: Figure 2(a) shows the Ta 3*d*_5/2_ spectra of the as-grown, formed and reset samples. The Ta 3*d*_5/2_ spectrum measured on the Al_2_O_3_/Ta interface outside the device areas is shown in the Supplementary Information (Fig. S2(a)). The spectrum measured outside the top electrode (Supplementary Fig. S2(a)) serves as a reference to distinguish the contribution of the Ta bottom electrode from the signal of the topmost TaN protective layer of the CBRAMs. The spectra have been fitted with three contributions. The first component is characteristic of metallic Ta at 1731.4 eV, the second component at 1733.4 eV is characteristic of TaN, only present in the capping layer, and tantalum sub-oxide (TaO_x_) and the third one at 1736.7 eV is attributed to Ta_2_O_5._ The Al_2_O_3_/Ta spectrum demonstrates that there is tantalum oxide at the Al_2_O_3_/Ta interface. The comparison between as-grown, formed and reset spectra showed a decrease of the metallic Ta contribution (− 6.4%) and an increase in oxidized Ta after forming and, conversely, an increase of metallic Ta (+12%) accompanied by a decrease in the TaO_x_ and Ta_2_O_5_ after reset (see Table 3).

**Table 3:** Relative areas (%) of the Ta 3*d*_5/2_ components measured outside the top electrode and for the as-grown, formed and reset samples.

	Ta	TaOx or TaN	Ta_2_O_5_
Al_2_O_3_/Ta	74.2 ± 0.2	13.3 ± 0.2	12.5 ± 0.2
As-grown	21.0 ± 0.2	41.0 ± 0.2	38.0 ± 0.2
Formed	14.6 ± 0.2	44.0 ± 0.2	41.4 ± 0.2
Reset	26.7 ± 0.2	39.7 ± 0.2	33.6 ± 0.2

Figure 2(b) presents the Ti 1*s* spectra which also have three contributions. The low binding energy (LBE) component at 4965.6 eV was fitted using a Doniach-Sunjic function^29^ and is characteristic of the metallic TiTe alloy. The second component at 4967.0 eV was fitted using a Lorentzian-Gaussian function and is characteristic of a TiO_x_ suboxide and the third one at 4968.7 eV is attributed to TiO_2_. From these spectra we conclude that the TiTe layer is oxidized at both TiTe/Al_2_O_3_ and TiTe/TaN interfaces.

The relative areas of each component for as-grown, formed and reset spectra are reported in Table 4. We observe a decrease of the TiO_2_ area (−9.4%) after forming. This is consistent with oxygen migration towards the Ta bottom electrode favoring TaO_x_ oxidation. On the other hand, the reset operation caused an increase of TiO_2_ (+11.6%), again consistent with oxygen transport back into the TiTe top electrode, favoring TaO_x_ reduction at the Al_2_O/Ta interface. Such a push-pull relationship has already been reported for OXRRAMs, see for example Berthaud *et al*.^31^. Furthermore a correlation between oxygen migration and applied voltage on the device has been shown previously for the ZrTe/Al_2_O_3_ stack under positive forming^9^.

**Table 4:** Relative areas (%) of the Ti 1*s* components for the as-grown, formed and reset samples.

	TiTe	TiO_x_	TiO_2_
As-grown	32.4 ± 0.1	27.2 ± 0.2	40.4 ± 0.1
Formed	32.5 ± 0.1	36.5 ± 0.2	31.0 ± 0.1
Reset	30.9 ± 0.1	26.5 ± 0.2	42.6 ± 0.1

Thus, the analysis of the Ti and Ta components of the top and bottom electrodes showed that the applied bias causes oxygen migration in the direction of the polarity, driving redox reactions at both top and bottom interfaces. Reduction of the pre-existing TiO_x_ at the TiTe/Al_2_O_3_ and TaN/TiTe interfaces occurs together with oxidation of TaO_x_ at the Al_2_O_3_/Ta interface for forming, consistent with downward oxygen migration. For the positive reset bias, the TiO_x_ is oxidized at the top interface while at the bottom electrode, TaO_x_ is reduced, consistent with upwards oxygen migration. These observations, while providing valuable insight into the oxygen push-pull process, do not reveal the filament formation in the resistive switching.

**Figure 3:**
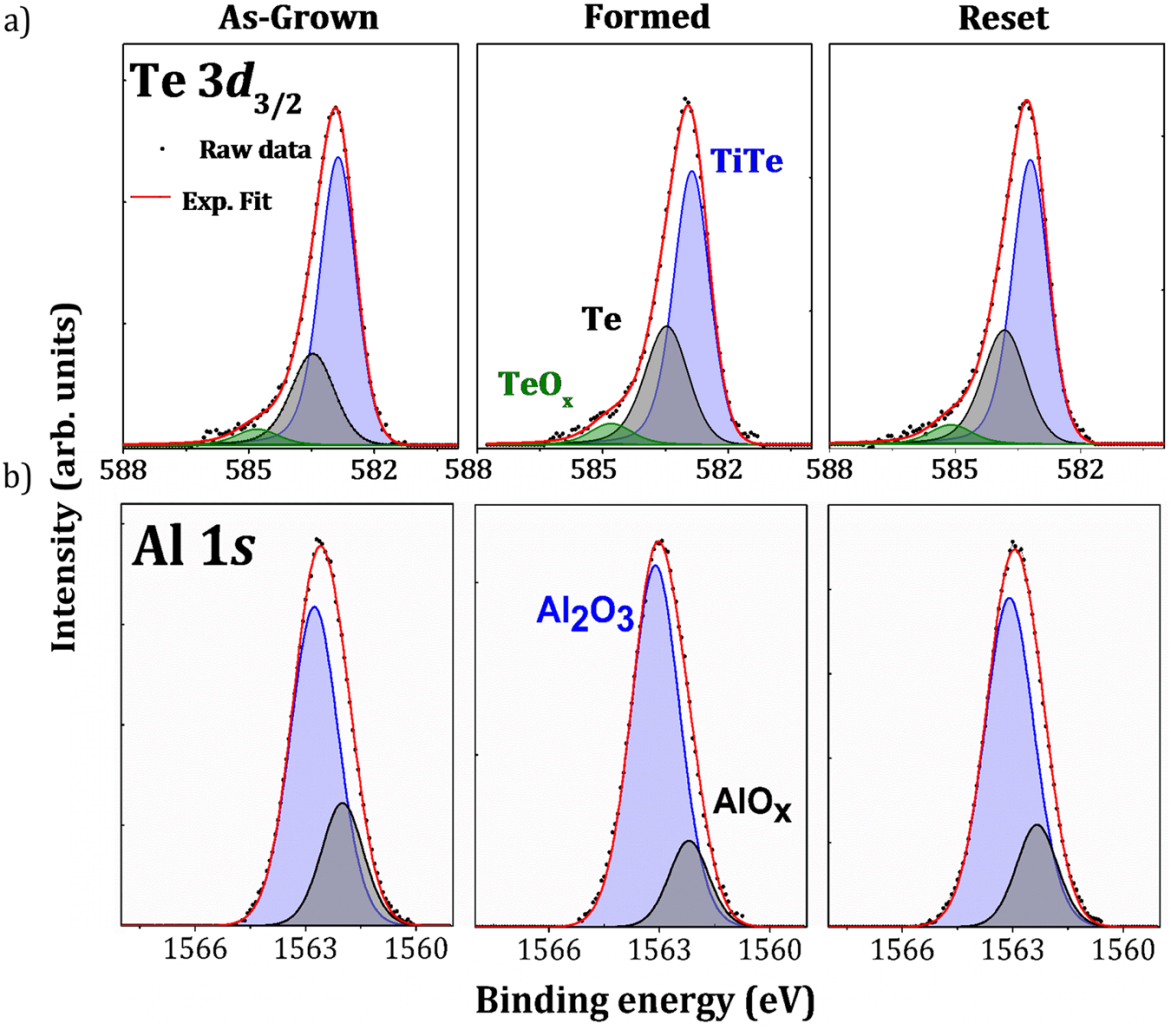
(**a**) Te 3*d*_3/2_ and (**b**) Al 1*s* core level peaks obtained by HAXPES at 6.9 keV on as-grown, formed and the reset sample of the TaN/TiTe/Al_2_O_3_/Ta stack.

Te and electrolyte chemistry: The Te 3*d*_5/2_ core level was not used in our measurements because of the overlap with the Ta *4*s core level. Figure 3(a) shows the Te 3*d*_3/2_ spectra. The top electrode is a metallic TiTe alloy but the Te spectra are asymmetric to higher binding energy, suggesting the possible presence of additional components. There are two possibilities for the HBE components: phase separation with formation of elemental Te (semiconductor) and/or oxidation of Te. The spectra have therefore been fitted with three components. The major component at 582.9 eV is related with the TiTe alloy using simple electronegativity arguments, the second component at 583.6 eV is characteristic of elemental semiconductor Te and the HBE component at 584.9 eV is related to the oxide (TeO_x_)^32,33^.

Table 5 shows the relative areas of each contribution in the as-grown, formed and reset spectra. After forming, a decrease in the Ti-Te environment (−7.2%) and an increase in elemental Te (+6.6%) was observed, whereas the reset causes a decrease of elemental Te (−2.7%) and an increase of the Ti-Te bonds (+2.3%). Importantly, both formed and reset samples show evidence for increased TeO_x_ coordination with respect to the pristine sample. This suggests a non-reversible correlation between the increase in elemental Te and oxygen environment.

**Table 5:** Relative areas (%) of the Te 3*d*_3/2_ components for the as-grown, formed and reset samples.

	TiTe	Te	TeO_x_
As-grown	69.1 ± 0.1	26.0 ± 0.3	4.9 ± 0.4
Formed	61.9 ± 0.1	32.6 ± 0.3	5.5 ± 0.4
Reset	64.2 ± 0.1	29.9 ± 0.3	5.9 ± 0.4

The Al 1*s* core level spectra are shown in Fig. 3(b). The reference spectrum measured outside the top electrode on the Al_2_O_3_/Ta is shown in Fig. S2(b) of the Supplementary Information. On the basis of the latter, the Al 1s spectra can be fitted using two components. The two contributions are a sub-oxide (labeled AlO_x_) at lower binding energy (BE) of 1561.9 eV and fully oxidized Al_2_O_3_ at a BE of 1562.7 eV. The relative areas are reported in Table 6.

**Table 6:** Relative areas (%) of the Al 1s components measured outside the top electrode and for the as-grown, formed and reset samples.

	Al_2_O_3_	AlO_x_
Al_2_O_3_/Ta	90.9 ± 0.2	9.1 ± 0.4
As-grown	74.7 ± 0.2	25.3 ± 0.4
Formed	83.7 ± 0.2	16.3 ± 0.4
Reset	79.5 ± 0.2	20.5 ± 0.4

The spectrum measured outside the top electrode (Supplementary Fig. S2(b)) showed the alumina reduction near the Ta bottom electrode, consistent with the presence of a TaO_x_ layer (Fig. S2(a)). Comparison between this spectrum and that of the as-grown sample showed an increase of the relative AlO_x_ contribution (+16.2%) in the latter. This result highlights the additional alumina reduction caused by the top electrode deposition. Similar to the case of a ZrTe top electrode^9^, oxygen scavenging is induced by Ti near the TiTe/Al_2_O_3_ interface. Pumping of oxygen by the Ti evidenced in Fig. 2b causes the creation of positively charged oxygen vacancies ${{\rm{V}}}_{{\rm{o}}}^{\bullet \bullet {}^{}}\,$in the electrolyte. This reduction of the electrolyte may play a significant role in the forming process^34–36^. Note that oxidation of the TiTe electrode may also occur through the surface by oxygen insertion in the TaN capping layer. This protective layer is thinned to 5 nm to enable HAXPES analyses of the buried TiTe/Al_2_O_3_ and Al_2_O_3_/Ta interfaces, may allow some oxygen transport from atmosphere.

The sub-oxide contribution (AlO_x_ at 1561.9 eV) decreases by 9% relative to the main contribution after forming. This is at first sight surprising since forming involves oxygen migration from top to bottom electrode but may be understood if the diffusion results in oxidation of the Al suboxide at the bottom interface. Conversely, application of a positive bias voltage to reset the HRS causes an increase of the AlO_x_ contribution (+4.2%) for the reset state compared with the formed sample, consistent with oxygen transport from the electrolyte towards the top electrode, i.e. oxygen scavenging at the top interface.

The HAXPES analysis at 6.9 keV therefore shows evidence for oxygen migration towards the bottom electrode during forming and concomitant redox reactions at both interfaces.

#### Depth profiles

**Figure 4:**
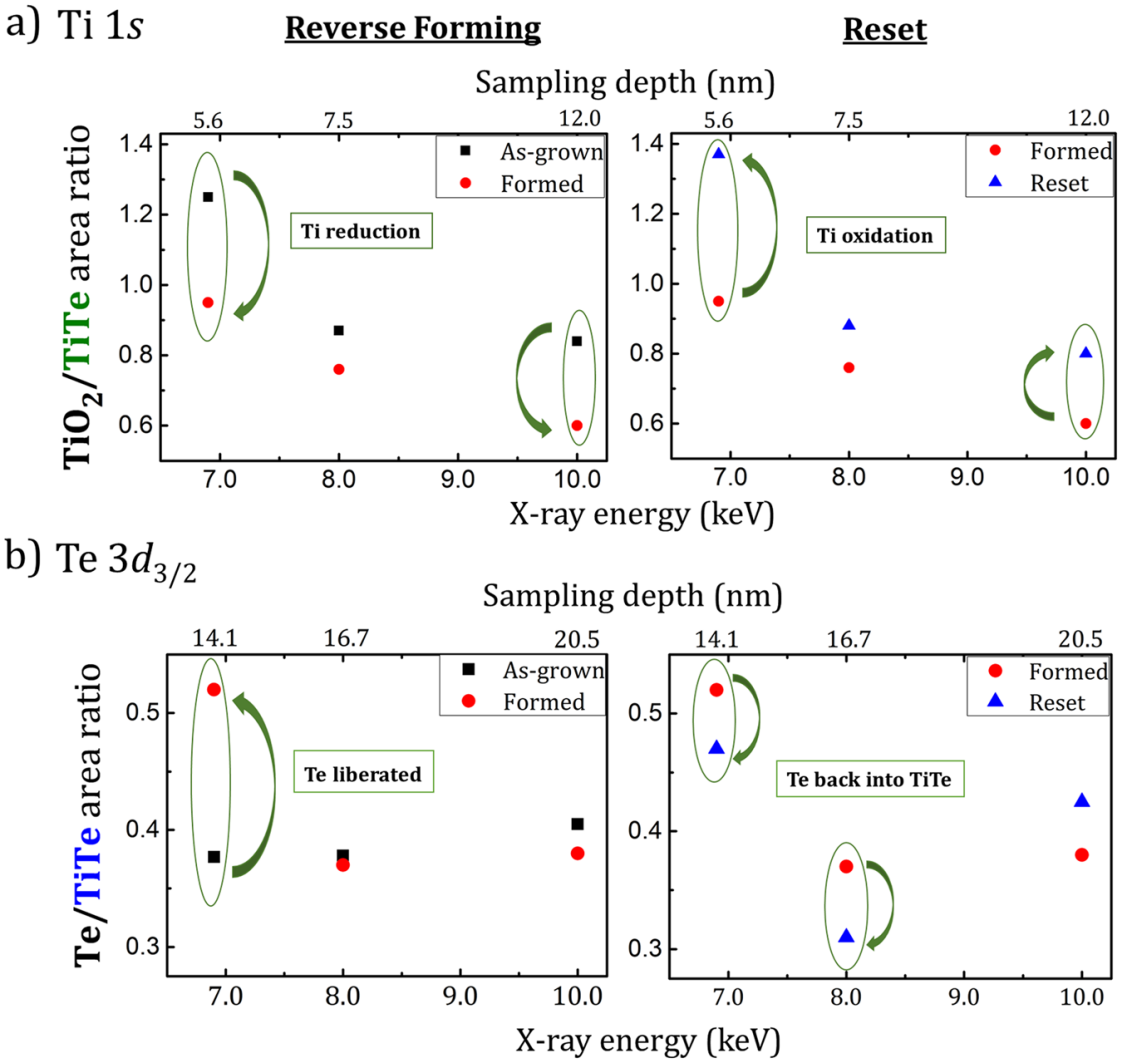
(**a**) TiO_2_/TiTe area ratios extracted from the Ti 1*s* line and (**b**) Te/TiTe area ratios extracted from Te 3*d*_3/2_ measured at 6.9, 8 and 10 keV for as-grown formed and reset samples.

We have performed the same analysis at 8 and 10 keV photon energy in order to vary the depth sensitivity (following the IMFP given in Table 1). Figure 4(a) shows the TiO_2_/TiTe area ratio extracted from the Ti 1*s* at 6.9, 8 and 10 keV for the forming and reset steps. During the forming, the TiO_2_/TiTe area ratio decreases at all the photon energies, indicating some reduction of the TiO_x_ interfacial layer. This reaction is related to the oxygen transport towards the alumina and the Ta bottom electrode, resulting in a decrease of the oxygen content in the TiTe layer. The opposite trend is observed for the reset at all the photon energies (increase of the TiO_2_/TiTe area ratio), showing the oxidation of TiO_x_. Oxygen is re-injected in the TiTe layer, under the effect of the positive bias. Changes are most pronounced at 6.9 keV, i.e. for a measurement more sensitive to the TiTe surface. The pre-existing TiTe oxidation through the capping layer is another reservoir for oxygen migration in the structure. The TiO_x_ reduction during forming and TiO_x_ oxidation during reset may thus also happen at the TaN/TiTe interface. We may also consider moisture at the sample surface as further oxygen reservoir. As reported by Tappertzhofen *et al*.^18^, OH^−^ ions may be supplied by the water reduction reaction and be injected in the structure during forming.

The Te/TiTe area ratios are plotted in Fig. 4(b) as a function of photon energy and sampling depth (1.5λ). On forming, the Te contribution is enhanced at 6.9 keV (1.5 λ = 14.1 nm) at the expense of the TiTe component whereas almost no changes are observed at higher energies (8 keV and 10 keV), more sensitive to regions deeper in the stack. Te is liberated in the TiTe electrode and accumulates near the interface with Al_2_O_3_. After the reset, there is a decrease of the Te/TiTe ratio for the lowest photon energies (6.9 keV and 8 keV), most sensitive to the TiTe/Al_2_O_3_ interface. We suggest that this may reflect Te partially driven back into the TiTe.

**Figure 5:**
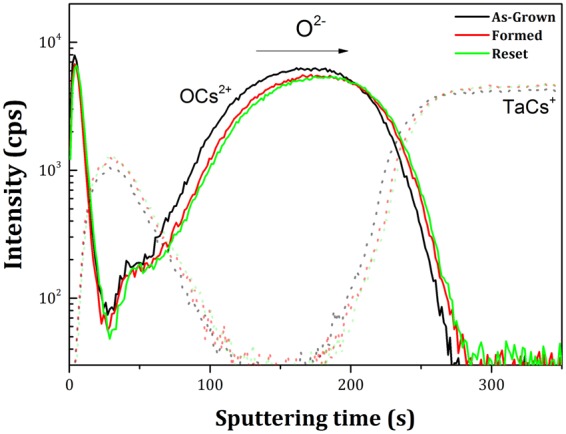
ToF-SIMS depth profiles of as-grown, formed and reset samples for OCs_2_^+^ and TaCs^+^.

In order to further determine the oxygen depth distribution, ToF-SIMS depth profiling was used. Figure 5 shows the oxygen and tantalum ToF-SIMS profiles in the MCs_n_^+^ mode (OCs_2_^+^ and TaCs^+^) for as-grown, formed and reset samples. The TaCs^+^ depth profiles (dotted lines) were used in order to guide the eye and define the top (TaN) and bottom (Ta) electrodes and thus to facilitate the analysis of the oxygen distribution in the stack. The depth profiles for the other elements present in the stack are shown in the Supplementary Information (Fig. S3).

The formed sample depth profile (red line) showed a shift of the oxygen content towards the bottom electrode in comparison to as-grown (black line). This shift is correlated with the HAXPES analysis that shows an oxygen movement from the upper negatively biased electrode towards the bottom electrode driven by the electric field. The variation in the oxygen content is not so pronounced for the reset sample in comparison to formed since the reset process causes smaller changes in the structure than the forming process^1,4^.

## Discussion

**Figure 6:**
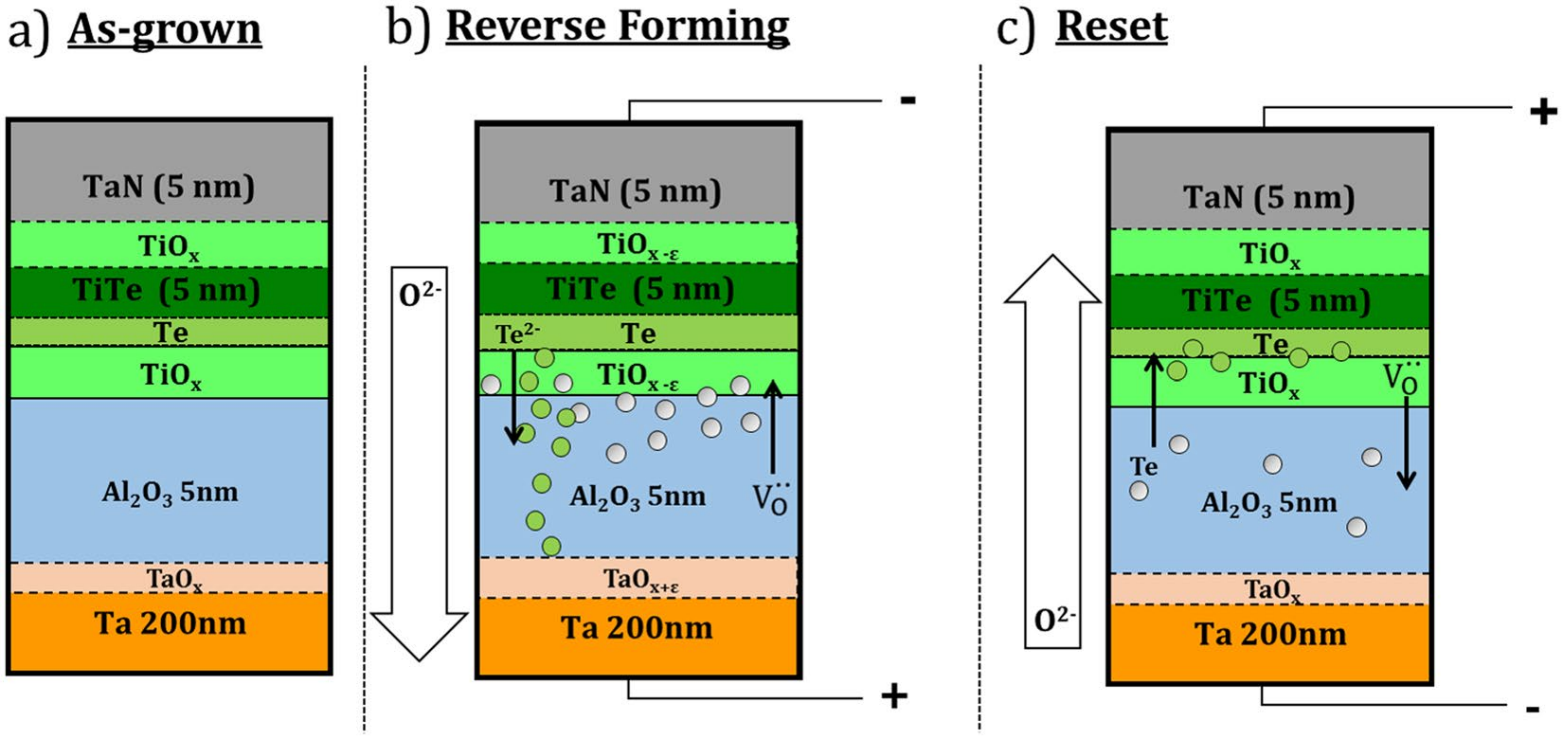
Schematic of the (**a**) as-grown sample and the redox processes and matter transport taking place during (**b**) forming and (**c**) reset in the TaN/TiTe/Al_2_O_3_/Ta stack.

Two distinct ion transport mechanisms appear to be at work during the forming and reset stages. The reduction of the pre-existing TiO_x_ layer at the TiTe/Al_2_O_3_ interface (see Fig. 4(a)) after forming is correlated with the variation of the oxygen distribution as a function of sampling depth, shown in Fig. 5. These results are consistent with an oxygen transport towards the bottom electrode and probably an increase of the ${{\rm{V}}}_{{\rm{o}}}^{\bullet \bullet {}^{}}$ concentration near the TiTe/Al_2_O_3_ interface as shown in Fig. 6. In turn, the oxidation of the alumina layer deduced from the decrease of the AlO_x_ component after the forming process (− 9%) shown in Fig. 3(b) may be related to the oxidation of the alumina layer near of the Al_2_O_3_/Ta interface caused by the diffusion of O^−2^ driven by the electric field.

The pre-existing TaO_x_ layer at the bottom electrode was also oxidized during the forming process, acting as an oxygen getter. Therefore the electrochemical reactions that occurs at each interface, based on these results, can be described as:

At the TiTe/Al_2_O_3_ interface:

1 $${{\rm{T}}{\rm{i}}{\rm{O}}}_{{\rm{x}}}+2{\rm{x}}\,{{\rm{e}}}^{-}\leftrightarrow {{\rm{T}}{\rm{i}}{\rm{O}}}_{{\rm{x}}/2}+{\rm{x}}/2\,{{\rm{O}}}^{2-}$$

At the Al_2_O_3_/Ta interface:

2 $${{\rm{T}}{\rm{a}}{\rm{O}}}_{{\rm{x}}/2}+{\rm{x}}/2\,{{\rm{O}}}^{2-}\leftrightarrow {{\rm{T}}{\rm{a}}{\rm{O}}}_{{\rm{x}}}+2{\rm{x}}\,{{\rm{e}}}^{-}$$

The reverse phenomena (TiO_x_ oxidation and TaO_x_ reduction) are observed during the reset, showing oxygen movement from Al_2_O_3_/Ta interface to TiTe layer. During the reset, the alumina is reduced at the Al_2_O_3_/Ta bottom interface due to oxygen migration towards the top electrode whereas oxygen is injected into the interface layer with the TiTe electrode which acts as a getter. The results are consistent with an oxygen transport across the stack, driven by the polarity of the applied bias. They suggest push-pull redox reactions happening at the interfaces with the top and bottom electrodes as a function of bias^31^. The key point is that the field induces matter transport and redox reactions at both top and bottom electrode/electrolyte interfaces. Thus, both top and bottom electrode should be considered as active with respect to oxygen transport (getters and reservoirs). These results denotes a pure OXRRAM mechanism with ${{\rm{V}}}_{{\rm{o}}}^{\bullet \bullet \,}$formation inside of the oxide by oxygen transport. As described by Molas *et al*.^36^, for a Cu-based cell, a pre-forming under negative polarization can be used to improve the RS of a CBRAM inducing oxygen vacancies in the oxide and facilitating the injection of Cu ions inside the electrolyte during the subsequent cycles of the cell.

A combination of both CBRAM and OxRRAM mechanisms is also possible^13,36^. The Te/TiTe intensity ratio analysis is consistent with significant Te release during forming and possible Te diffusion. The intensity ratio suggests an accumulation of elemental tellurium at the TiTe/Al_2_O_3_ interface after the forming process (see Fig. 4(b)). Te release was favored by the oxidation of Zr in the case of standard forming of ZrTe-based CBRAMs^9^. Here, however, the mechanism may be different because TiO_x_ is reduced at the TiTe/Al_2_O_3_ interface because of oxygen transport during the forming whereas the Ti metal peak intensity is unchanged. The driving force for Te migration might be the electric field, pushing Te^2−^ towards the bottom electrode. Indeed, Te is known to be negatively ionized in TiTe or ZrTe alloys because of the higher Te electronegativity (2.1) than Ti/Zr (1.6/1.5). This is consistent with the increase of Te in the elemental form in the Te 3*d*_3/2_ spectra and the slight decrease after resetting. Te which has separated out at the interface or even migrated inside Al_2_O_3_ is also more likely to have an environment including some oxygen atoms, which could well explain the increase in the TeO_x_ component after forming. The major changes in the Te intensities happen during forming and are not recovered during the reset, evidence that the forming process is not fully reversible and that Te does indeed participate in the resistive switching.

On the basis of these results, we suggest the forming and reset mechanisms which are shown schematically in Fig. 6. The layer structure is only a schematic to allow approximate model calculations of equivalent thicknesses. For example, the interface layer of the top electrode could well be a phase separated TiO_x_, Te and TiTe. The effective layer thicknesses calculated here should therefore be treated as (quite good) approximations. To quantify these phenomena, we have used a multilayer model^9,37^ to study in detail the thickness variation of the elemental Te and TiO_2_ layers for as-grown, formed and reset sample (see Supplementary Information Fig. S4 and Table S1).

During forming, we think there is coexistence of interfacial redox processes and localized diffusion mechanisms. Redox processes take place at both TiTe/Al_2_O_3_ and Al_2_O_3_/Ta interfaces. TaO_x_ at the bottom electrode is oxidized, with a correlated TiO_x_ reduction at the top electrode. These redox processes are activated by oxygen migration, the O^2−^ ions drifting downwards under the negative bias applied on the top TaN electrode and a corresponding increase of the oxygen vacancies concentration at the TiTe/Al_2_O_3_ interface. In parallel, the HAXPES spectra highlight the accumulation of Te at the TiTe/Al_2_O_3_ interface. Similarly to oxygen, Te^2−^ might also drift inside the alumina under the applied field. Considering a hybrid mechanism, a possible physical interpretation of the forming mechanism is thus the coexistence of phenomena typical of both CBRAMs (Te migration) and OXRRAMs (${{\rm{V}}}_{{\rm{o}}}^{\bullet \bullet \,}$formation by oxygen transport). Thus, the resistive switching mechanism is possibly related to the presence of ${{\rm{V}}}_{{\rm{o}}}^{\bullet \bullet \,}$ inside the alumina but also to Te drift^12–14^. Such hybrid mechanisms have recently been reported in several papers. Several authors suggest that the formation of metallic filaments is helped by oxygen vacancies, favoring cation insertion inside the electrolyte under bias application^18,38^. Other studies have highlighted conducting paths made by a combination of metallic filaments (Cu, Ag) and oxygen vacancies^35,39,40^.

The ions and ${{\rm{V}}}_{{\rm{o}}}^{\bullet \bullet \,}$ transport mechanisms are reversed during the reset a) TaO_x_ reduction and TiO_x_ oxidation following the oxygen movement towards the top electrode, b) less free Te is observed probably because driven back into the top electrode, reforming the TiTe alloy. Both the recombination of oxygen ions with ${{\rm{V}}}_{{\rm{o}}}^{\bullet \bullet \,}$ at the TiTe/Al_2_O_3_ interface by reoxidation and the loss of Te near TiTe might help to switch back the structure in a high resistance state.

This work provides evidence that the OXRRAM mechanism occurs in the TiTe/Al_2_O_3_-based CBRAM when a negative voltage is applied on the device. In addition the results showed a Te release from the top electrode during the forming process. Note that the increase of the elemental Te does not necessarily imply migration, it may also be due to the formation of Te-Te bonds inside the top electrode. In this case only the formation of oxygen vacancies inside of the electrolyte then an OXRRAM behavior may explain the resistive switching of the forming process.

## Conclusion

In summary, it can be concluded that in the reverse forming of Te-based subquantum CBRAMs the Ta bottom electrode acts as an oxygen getter creating oxygen vacancies at the TiTe/Al_2_O_3_ interface. The oxygen movement in the form of O^2−^ is driven by the electric field from the upper negatively biased electrode towards the bottom one. Forming also causes an accumulation of elemental Te near the top interface. Te^2−^ might also be pushed inside the alumina by the electric field. On reset there is a partial recombination of oxygen ions with ${{\rm{V}}}_{{\rm{o}}}^{\bullet \bullet \,}$ near TiTe/Al_2_O_3_ interface together with a loss of Te. These results strongly suggest that CBRAM (Te transport) and OxRRAM (${{\rm{V}}}_{{\rm{o}}}^{\bullet \bullet \,}$ migration) mechanisms coexist in these Te-based memory cells. Future experiments should include bringing an experimental proof of possible conductive paths in Al_2_O_3_ and cycling between set and reset states in order to study the chemistry of cell endurance.

## Electronic supplementary material

Supplementary information

## Data Availability

The datasets generated and/or evaluated during the current study are available from the corresponding author on reasonable request.
