# Supplementary material for: Chemistry of resistivity changes in TiTe/Al2O3 conductive-bridge memories
Source: Sci Rep. 2018 Dec 18;8:17919. doi: 10.1038/s41598-018-36131-7 (PMC6298955; doi:10.1038/s41598-018-36131-7)
Supplement: Supplementary file 1 — Supplementary information [file 41598_2018_36131_MOESM1_ESM.docx]

Supplementary Information for

**Chemistry of resistivity changes in TiTe/Al_2_O_3_ conductive-bridge memories**

**M. Kazar Mendes^1*^, E. Martinez^1^, J.M. Ablett^2^, M. Veillerot^1^, R. Gassilloud^1^, M. Bernard^1^, O. Renault^1^, J. P. Rueff^2, 3^, and N. Barrett^4^**

^1^ Univ. Grenoble Alpes, CEA, LETI, 38000 Grenoble, France

^2^Synchrotron SOLEIL, l’Orme des Merisiers, Saint-Aubin, F-91192 Gif-sur-Yvette Cedex, France

^3^Sorbonne Université, UPMC Univ Paris 06, CNRS, UMR 7614, Laboratoire de Chimie Physique-Matière et Rayonnement, 75005 Paris Cedex 05, France

^4^SPEC, CEA, CNRS, Université Paris-Saclay, CEA Saclay, 91191 Gif-sur-Yvette, France

*) Author to whom correspondence should be addressed.

Electronic mail: [Munique.KAZARMENDES@cea.fr](mailto:Munique.KAZARMENDES@cea.fr)

**Supplementary Note 1.** IMFPs estimation

The IMFPs were estimated with the Tanuma equation ^1^. These values were obtained by averaging the IMFPs estimated for each layer crossed by the photoelectrons during their transport toward the surface. The average was weighted by the thickness of each layer. For example the relation below shows the estimation of the $for Al 1s \mathrm{photoelectrons}$considering a stack of TaN/TiTe/Al_2_O_3._

${}_{Al 1s} =\frac{\left[ \left( {}_{Al, TaN}\times d_{TaN} \right)+\left( {}_{Al, TiTe}\times d_{ZrTe} \right)+\left( {}_{Al, {Al}_{2}O_{3}}\times d_{{Al}_{2}O_{3}} \right) \right]}{\left( d_{TaN}+ d_{TiTe}+ d_{{Al}_{2}O_{3}} \right)}$ (S1)

where ${}_{Al, TaN}$ , ${}_{Al, TiTe} \mathrm{and} {}_{Al, {Al}_{2}O_{3}}$ are the IMFPs estimated in the TaN , TiTe and Al_2_O_3_ layers respectively as well as $d_{TaN}, d_{TiTe} and d_{{Al}_{2}O_{3}}$are the thickness of each layer.

**Supplementary Note 2.** Electrical characterization of the formed sample:


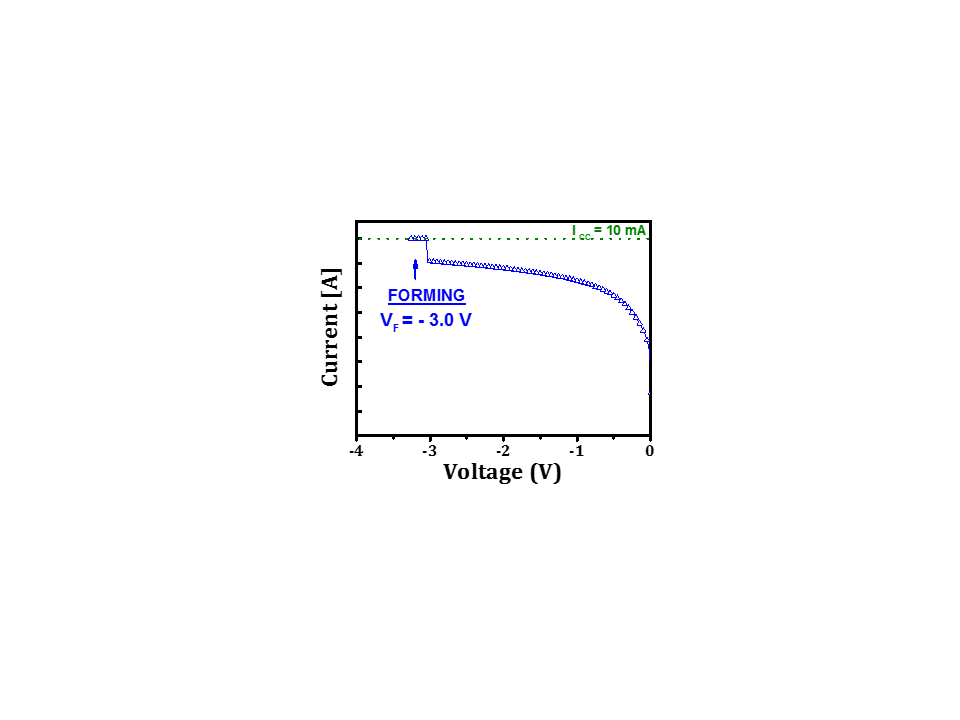


**Figure S1.** Current - voltage (I-V) curve for the forming of the TaN/TiTe/ Al_2_O_3_/Ta structure.

Figure S1 presents the current - voltage (I-V) curve for the forming process of the called formed sample. The device resistances measured before and after forming are equal to R_As-grown_ = 1.0 10^5^ Ω and R_Formed_ = 1.3 10^2^ Ω. The corresponding resistance ratio is R_As-grown_ / R_Formed_ = 8 10^2^ Ω.


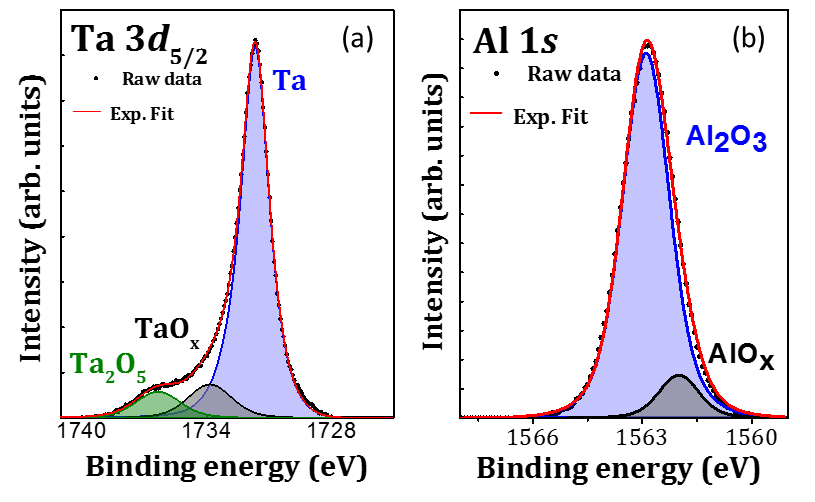


**Figure S2**. (a) Ta 3*d*_5/2_ and (b) Al 1*s* core level spectra obtained at 6.9 keV photon energy on the bare Al_2_O_3_/Ta structure.

The Ta 3*d*_5/2_ and Al 1*s* core level spectra were also measured on the bare Al_2_O_3_/Ta structure outside the top electrode to provide reference spectra for tantalum and aluminum (see Figure S2). The Ta 3*d*_5/2_ spectrum is characteristic of metallic Ta (1731.4 eV). Two oxide contributions (TaO_x_ at 1733.4 eV and Ta_2_O_5_ at 1736.7 eV) indicate the presence of Ta oxide at the Al_2_O_3_ interface. The two contributions to the Al 1s core level spectra are a sub-oxide (labeled AlO_x_) at lower BE of 1561.9 eV and alumina at BE of 1562.7 eV.

**Supplementary Note 3. ToF-SIMS depth profiles**

**
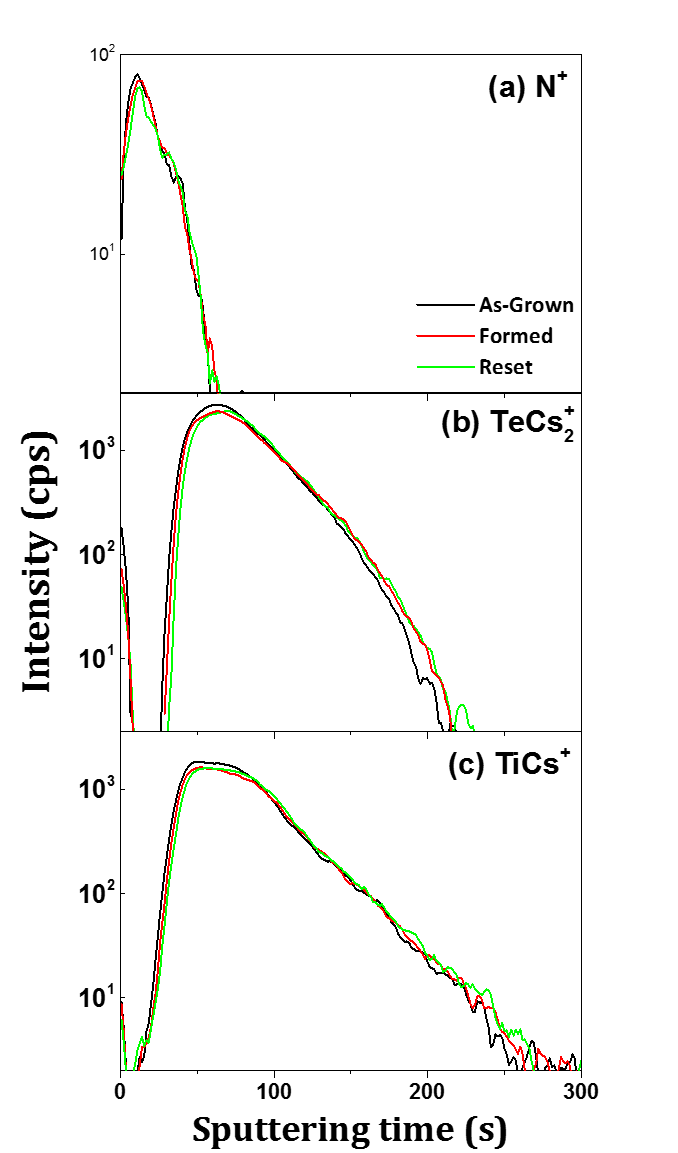
**

**Figure S3.** ToF-SIMS profiles of as-grown, formed and reset samples for N^+^, TeCs_2_^+^ and TiCs^+^.

Figure S3 presents the (a) nitrogen, (b) tellurium and (c) titanium ToF-SIMS profiles in the MCs_n_^+^ mode (TeCs_2_^+^, TiCs^+^) and M^+^ mode (N^+^) for as-grown, formed and reset samples. The N^+^ and TiCs^+^ profiles are rather stable for the three resistive states, confirming the reliability of the measurements. The TeCs_2_^+^ profiles measured after forming and reset are slightly shifted towards the bottom electrode, confirming the possible Te diffusion in the electrolyte. The major changes happen again during forming, confirming that the initial state is not completely recovered during the reset.

**Supplementary Note 4. Multilayer model**

To try to quantify the redox process caused by the forming and reset processes, we have used a multilayer model ^2,3^ . We assume the presence of distinct layers of TiO_2_ and Te at the TiTe/Al_2_O_3_ upper interface, as shown in Fig. 6 and Fig. S4. This is clearly a simplification, since any redox induced phase separation may also happen to some extent within the same layer. We also assume that the released Te mainly stays at the interface. To calculate their respective thicknesses, the intensity ratios extracted from the Ti 1*s* and Te 3*d*_3/2_ core levels can be expressed using the equation (1) and (2) as detailed in ^2^. We assume that d_TiO2_+ d_TiTe_ = 5 nm and d_Te_+ d_TiTe_ = 5 nm and that the IMPFs of Ti 1*s* and Te 3*d*_3/2_ photoelectrons were the same in all the layers of the stack and equal to λ_Ti_ = 3.7 nm and λ_Te_ = 9.8 nm, respectively. This leads to an uncertainty of ± 0.7 nm in the elemental Te layer estimation.

$\frac{I_{\begin{aligned} TiO2 \\ \end{aligned}}}{I_{\begin{aligned} TiTe \\ \end{aligned}}}= \frac{I_{TiO2}}{I_{TiTe}}\exp\left( \frac{- d TiTe}{\mathrm{Ti}} \right)\frac{1-\exp\left( \frac{-d TiO2}{Ti} \right)}{\left[ 1-\exp\left( \frac{-d TiTe}{Ti} \right) \right]}$ (1)

$\frac{I_{\begin{aligned} Te \\ \end{aligned}}}{I_{\begin{aligned} TiTe \\ \end{aligned}}}= \frac{I_{Te}}{I_{TiTe}}\exp\left( \frac{- d TiTe}{\mathrm{Te}} \right)\frac{1-\exp\left( \frac{-d Te}{Te} \right)}{\left[ 1-\exp\left( \frac{-d TiTe}{Te} \right) \right]}$ (2)

The Ti 1*s* and Te 3*d*_3/2_ spectra measured at 6.9 keV have been used to estimate respectively the TiO_2_ and Te thicknesses, because the changes are more pronounced at this energy. The results obtained for the reverse forming and the reset are summarized in Fig. S4 and Table S1.


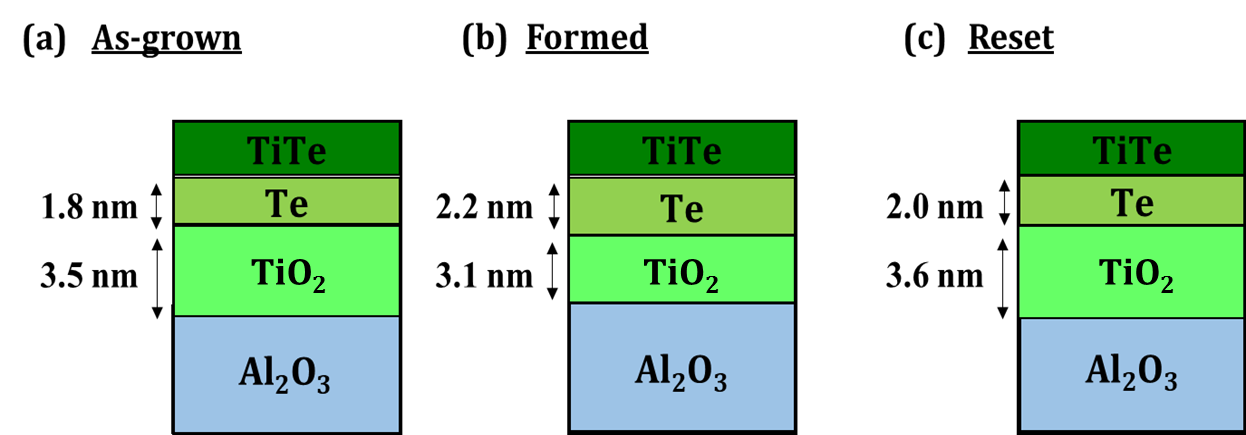


**Figure S4.** Evolution of the thicknesses of the Te and TiO_2_ layers during reverse forming and reset in the TaN/TiTe/Al_2_O_3_/Ta memory stack.

TABLE S1. Experimental intensity ratios and estimated thicknesses of the TiTe/Te and TiTe/TiO_2_ stacks extracted from the quantitative model and the Te 3*d*_3/2_ and Ti 1*s* core-level components intensities.

| Sample | I_TiO2_/I _TiTe_ | I _Te_ / I _TiTe_ | d TiO_2_  (nm) | d Te  (nm) |
| --- | --- | --- | --- | --- |
| As-grown | 1.26 | 0.37 | 3.5 | 1.8 |
| Formed | 0.95 | 0.52 | 3.1 | 2.2 |
| Reset | 1.37 | 0.46 | 3.6 | 2.0 |

After the reverse forming, we see an increase of the interfacial Te-rich layer and a decrease of TiO_2_ layer thicknesses of the same order of magnitude (~0.4 nm). This result agrees well with the previous conclusions regarding the accumulation of Te and the accumulation of oxygen vacancies in the TiTe/Al_2_O_3_ interface. Both phenomena seem to contribute in the same order of magnitude to the resistive switching mechanism. After the reset, the Te-rich layer thicknesses decrease and the TiO_2_ layer thicknesses increase of 0.2 nm and 0.5 nm respectively. The dissolution of oxygen vacancies seems to be the major mechanism breaking the conductive paths.

**References**

1. Tanuma, S., Powell, C. J. & Penn, D. R. Calculations of electron inelastic mean free paths. V. Data for 14 organic compounds over the 50–2000 eV range. *Surf. Interface Anal.* **21,** 165–176 (1994).

2. Mendes, M. K. *et al.* Forming mechanism of Te-based conductive-bridge memories. *Appl. Surf. Sci.* **432,** 34–40 (2018).

3. Jablonski, A. & Zemek, J. Overlayer thickness determination by XPS using the multiline approach. *Surf. Interface Anal.* **41,** 193–204 (2009).
